# SKAP1 is dispensable for chemokine-induced migration of primary T-cells

**DOI:** 10.1016/j.imlet.2009.10.010

**Published:** 2010-02-16

**Authors:** Hongyan Wang, Yuning Lu, Christopher E. Rudd

**Affiliations:** a Department of Pathology, University of Cambridge, Tennis Court Road, Cambridge CB2 1QP, UK; b Cambridge Institute for Medical Research, Addenbrooke's Hospital, Hills Road, Cambridge CB2 0XY, UK

**Keywords:** SKAP1, Chemokine, T-cell migration

## Abstract

Immune adaptors SLP-76, ADAP and SKAP1 (SKAP-55) play central roles in anti-CD3 induced ‘inside-out’ signalling for LFA-1 activation and ICAM-1 adhesion. However, it has been unclear whether SKAP1 is also required for chemokine-induced T-cell motility. In this study, we found that SDF-1 and CCL21 induced similar motility in SKAP1 deficient (*SKAP1*−/−) and wild type (SKAP1+/+) resting, primary T-cells. In addition, the speed (i.e. 13 μM/min), tracking distance (i.e. length) and displacement values (i.e. direct distance between the start and the end positions of cell movement) in response to SDF1 were similar for *SKAP1*−/− and SKAP1+/+ primary, activated T-cells. Relatively high strength anti-CD3 ligation also arrested the migration (i.e. stop-signal) of resting SKAP1+/+ and *SKAP1*−/− T-cells in the presence of SDF-1 and CCL21. These data demonstrate that contrary to its central role in anti-CD3 induced LFA-1 adhesion, the response of primary T-cells to SDF-1 and CCL21 is not profoundly dependent on SKAP1 expression.

## 1 Introduction

Chemokines are chemo-attractants of 7–10 kDa that are named based on the number and spacing of cysteine residues in the amino-terminal region as C, CC, CXC or CX3C. They regulate multiple cellular functions, including cell adhesion, proliferation, lymphopoiesis and cell movement to the sites of infection and inflammation [1]. Upon binding to chemokine receptors, chemokines generate signals that activate integrins, enabling T-cells to transmigrate through high endothelial venules (HEVs) lined in the blood vessel to sites of inflammation [2–4].

Integrins are transmembrane adhesion molecules that mediate cell–cell or cell–extracellular matrix adhesion. T lymphocytes express mainly β1, β2 and β7 integrins, especially αLβ2 (LFA-1: leukocyte function associated antigen-1) and α4β1 (VLA-4: very late antigen 4). In naïve T-cells, integrins are kept in inactive state, while the T-cell receptor (TCR) can induce ‘inside-out’ signals that activate integrins, partly due to increased LFA-1 clustering [5–7]. Chemokines can also generate intracellular signals that alter the affinity and avidity of LFA-1 function. Chemokines such as CCL2, CCL3, CCL4, CXCL10, CXCL9, CCL5, CXCL12 (also termed SDF-1, stromal cell-derived factor-1), CCL19, CCL21, CCL20, CCL17 and CCL22 are able to induce the activation of β1, β2 and β7 integrin-dependent adhesion [1]. The arrest in the motility of naïve T-cells in HEVs depends on integrins LFA1 and α_4_β_7_ as well as chemokines CCL21 and CXCL12/SDF-1. Chemokine receptors are seven-transmembrane-domain G-protein-coupled receptors (GPCRs) that can be divided into different groups, such that CCR7 binds CCL21 and CXCR4 binds CXCL12/SDF-1 [1].

Several signalling proteins including PI 3K (phosphoinositide 3-kinase) [8], GTPases RhoA [9], protein kinase C [9] and Rap1 [10], as well as the Rap1 ligand RapL (regulator of cell adhesion and polarisation enriched in lymphoid tissues) [11] play roles in chemokine signalling for integrin activation and migration. Rap1 and RapL also participate in anti-CD3 induced ‘inside-out’ signalling for integrin activation [12,13]. Studies by ourselves and others have identified immune cell specific adaptor proteins ADAP (adhesion and degranulation-promoting adaptor protein) and SKAP1 (src kinase-associated phosphoprotein 1: HUGO official designation; also SKAP-55, src kinase-associated phosphoprotein-55) that positively regulate TcR induced β1 and β2 integrin activation [14–18]. T-cells from *SKAP1*−/− mice show defective anti-CD3 induced LFA-1 clustering despite the presence of intracellular ADAP [17–18]. ADAP binds SKAP1 [19,20] and controls SKAP1 expression levels in T-cells [21]. One model reported that the ADAP–SKAP1 module controls Rap1–GTP membrane localisation [22], while SKAP1 directly interacts with the Rap1–GTP binding partners Riam [23]. In another model, SKAP1 expression is needed for anti-CD3 induced RapL membrane association and Rap1–RapL complex formation (unpublished data).

Although ADAP over-expression has been reported to increase CXCL12/SDF-1α induced cell migration in a Jurkat cell line [24], it is not known whether SKAP1 plays any role in chemokine-induced migration of primary T-cells. To address this question, we used resting primary T-cells from *SKAP1*−/− mice to assess motility in response to CXCL12 and CCL21 using dual chamber transwell assays. Surprisingly, neither speed nor direct movement to chemokine gradients was affected by the loss of SKAP1. Unlike in the case of anti-CD3 induced ‘inside-out’ signalling, SKAP1 is dispensable for both CXCL12 and CCL21 induced T-cell migration.

## 2 Material and methods

### 2.1 Reagents

CXCL12/SDF-1α and CCL21 was purchased from R & D System. Transwell plates were from Corning incorporated (Costar, Boston, MA, USA). Anti-mouse CXCR4 antibody conjugated to FITC and anti-mouse CD4 conjugated to APC were from BD. Flow-chamber plates were ordered from Ibidi (Munich, Germany).

### 2.2 Cell isolation and culture

Spleens were isolated from wild type or SKAP1 deficient mice meshed through cell strainers, followed by removal of red blood cells (RBC) with hypotonic buffer (0.15 M NH_4_Cl, 1 mM NaHCO_3_, 0.1 mM EDTA, PH 7.25). Splenocytes were supplemented with 10% foetal calf serum (FCS), 5% glutamine, 5% Pen-strep and 2-mercaptoethanol (2-ME) at 5 × 10^−5^ M. CD4^+^ T-cells were purified using mouse anti-CD4 coated Dynal beads (BD Biosciences, Oxford, UK). Alternatively, splenocytes were cultured with Con A (2 μg/ml) for 2 days, washed once with growth medium, and cultured with interleukin 2 (IL-2; 20 ng/ml) for 2–3 days to generate T-cell blasts. After washing residual IL-2, T-cells were rested in growth medium for 2 days, and were then used for experiments.

### 2.3 Chemotaxis assay

Chemokine-induced cell migration was assessed using 5-μm pore size transwells. The membrane inserts were placed in the wells of a 24-well plate, containing 600 μl buffer (RPMI with 0.5% FCS) with or without chemokines SDF-1 and CCR27 (100 ng/ml). 0.2 × 10^6^ freshly isolated T-cells or splenocytes in 100 μl buffer were loaded into each transwell filter. After incubation at 37 °C for various time courses, migrated cells to the bottom wells were collected and counted.

To measure TcR induced ‘stop’ signal, the membrane inserts were pre-coated with anti-CD3 antibodies (5 μg/ml) with or without ICAM-1 (5 μg/ml) for 2 h, washed once with PBS, then used for chemotaxis assay. Alternatively, cells were incubated with soluble anti-CD3 (5 μg/ml) for 10 min at 37 °C then loaded to transwell filters. To measure directional cell migration, cells were loaded in one well in the flow-chamber plate and chemokines were added in another well. Time lapse was used to monitor cell migration in the middle area between the two wells. Images were recorded every 5 s for 120 cycles. Cell tracks were analysed by Velocity Software.

### 2.4 Surface CXCR4 expression levels by flow cytometry

10^6^ resting or anti-CD3 stimulated WT or *SKAP1*−/− T-cells were incubated with 2 μl FITC-conjugated anti-mouse CXCR4 antibody or isotype control antibody in 200 μl PBS on ice for 40 min followed by washing once with PBS. The percentage or intensity of CXCR4 staining was analysed by BD FACSCalibur.

### 2.5 Statistical analysis

Results are given as the mean ± standard deviation (S.D.). A *P* value was tested using unpaired student *t*-test using GraphPad Prism version 3.02 (GraphPad Software, San Diego, California, U.S.A.) and *p* < 0.05 was considered as significant.

## 3 Results and discussion

### 3.1 SKAP1 is dispensable for SDF-1 induced resting T-cell migration

**Fig. 1:**
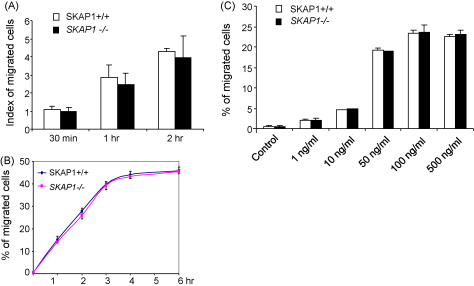
*SKAP1*−/− T-cells show normal SDF-1 induced cell migration (A–C). Panel A: Freshly isolated CD4^+^ T-cells (0.2 × 10^6^ in 100 μl RPMI with 0.5% FCS) from SKAP1+/+ and *SKAP1*−/− mice were added to the upper wells of the transwell plates and SDF-1 (100 ng/ml) was added to lower chambers containing 600 μl RPMI with 2% FCS. Cells were incubated at 37 °C for different time courses and the migrated cells to the lower chambers were harvested and counted. *SKAP1*−/− T-cells to WT cells, the *P* values in (A) are 0.47 (i.e. 30 min), 0.44 (1 h) and 0.71 (2 h). Panel B: Time course (0–6 h) of movement of SKAP1+/+ and *SKAP1*−/− T-cells to SDF1 in transwell plates. Panel C: Response to T-cells to different concentrations of SDF1. Different concentrations of SDF-1 were added to the bottom wells and cells that migrated to the bottom wells were counted after incubation for 2 h (*P* > 0.05, when compared SKAP1 KO samples with WT samples).

SKAP1 expression is needed for TcR induced ‘inside-out’ signalling for integrin activation in T-cells [16–18,22,23]. It has not been clear whether SKAP1 expression is also needed for chemokine-induced motility. Previous studies have clearly implicated Rap1–RapL in anti-CD3 and chemokine induced LFA-1 activation [12,13]. To address this, we initially examined whether SKAP1 could regulate the chemotaxis of resting, primary T-cells to SDF-1 and CCL21. For this, freshly purified resting SKAP1+/+ or *SKAP1*−/− CD4^+^ T-cells were seeded onto the upper well of a transwell plate, while SDF-1 was added to the lower well. After incubation for different times, cells that migrated to the lower well were counted. An increase in cell number in the lower chamber was observed over the time course. By 30 min, 5–8% of SKAP1+/+ cells had migrated to the lower well that increased to 12–15% by 1 h (Fig. 1A and B). After a 3–4-h incubation, the level of migration reached a plateau of 40–45% of cells (Fig. 1B). Surprisingly, a comparable number of wild type and *SKAP1*−/− cells migrated to the lower wells at all time points measured (Fig. 1A and B). To assess whether differences might become evident in the presence of different concentrations of SDF1, a titration was conducted with 1–500 ng/ml of chemokine over an incubation period of 2 h. At each concentration, equal numbers of resting SKAP1+/+ and *SKAP1*−/− T-cells migrated to the lower chamber (Fig. 1C). Occasionally, *SKAP1*−/− T-cells showed a slightly lower level of migration; however, the difference was less than 10% relative to WT cells and was not reproducible. Assays were performed in the presence of 0.5% FCS, although the same results were obtained in the absence of FCS (data not shown). These data showed that SKAP1 was not needed for resting T-cell migration to SDF-1 as determined by a transwell assay.

### 3.2 SKAP1 is dispensable for SDF-1 induced directional movement of activated primary T-cells

**Fig. 2:**
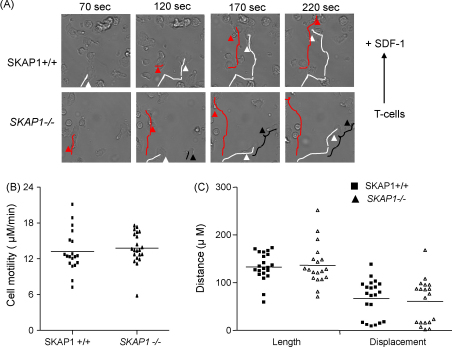
SKAP1+/+ and *SKAP1*−/− activated T-cells migrate at the same speed and distance in response to SDF1. T-cells were plates in one well in a flow-chamber plate and SDF-1 was added to another well. Time lapse was used to record cell movement every 5 s for 120 cycles. Representative images at the adjusted time points (A) and motility (B) and the distance of cell movement (C) were analysed by Velocity software.

Given this finding, we next assessed the movement of activated primary T-cells using transwell assays. However, this approach produced a high background migration of activated cells (data not shown). We therefore attempted to visualise SDF-1 induced directional T-cell migration on a horizontal interconnected flow chamber. One well containing T-cells was separated by a septum connected to another well with SDF-1. This allowed the establishment of a chemokine gradient between wells and the monitoring of directional cell migration every 5 s for 120 cycles using the time-lapse microscopy. Some 40% of SKAP1+/+ and *SKAP1*−/− T-cells migrated towards SDF-1 with representative images shown in Fig. 2A. Velocity software provided an objective measure of motility showing that both populations of T-cells migrated with an average speed of 13–15 μM/min (Fig. 2B). The length (whole distance following cell tracks) and displacement (direct distance between the start and the end points of cell movement) was also measured using the same software (Fig. 2C). Both SKAP1+/+ and *SKAP1*−/− T-cells migrated a distance of 140–145 μM over the time course and the two populations showed no significant difference in the displacement from the point of origin. These findings demonstrate that the absence of SKAP1 does not influence speed, or migration to SDF-1.

### 3.3 Anti-CD3 arrests the motility of *SKAP1*−/− T-cells in the presence of SDF1

**Fig. 3:**
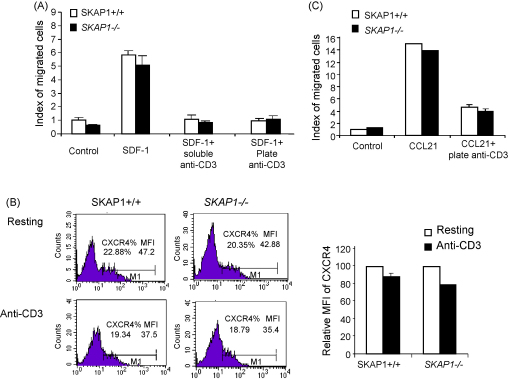
Anti-CD3 stimulation induces ‘stop-signal’ to inhibit SDF-1 or CCL21 induced cell migration in *SKAP1*−/− T-cells. Panel A: The transwell membrane was coated with anti-CD3 (5 μg/ml) in the presence or absence of ICAM-1 (5 μg/ml) for 2 h. After washing once with PBS, WT cells and *SKAP1*−/− T-cells were seeded to the upper wells and SDF-1 (100 ng/ml) was added to the bottom wells. The number of cells migrated over a 2 h period of exposure to medium alone, SDF-1 alone, or a combination of both SDF-1 and 2C11, were collected and counted. The *P* values are 0.0254 (Medium alone), 0.0877 (SDF-1), 0.1546 (SDF-1 + anti-CD3). Panel B: Fresh or anti-CD3 stimulated WT or SKAP1 KO CD4^+^ T-cells were stained with FITC-conjugated anti-CXCR4 and analyzed by FACS. The percentage of CXCR4 positive cells and the mean fluorescence intensity of CXCR4 staining were provided in the FACS profiles. The histogram shows the relative CXCR4 expression levels in anti-CD3 stimulated cells compared to those in resting cells. Panel C: SKAP1+/+ cells and *SKAP1*−/− T-cells were seeded in the upper wells and CCL21 (100 ng/ml) was added to the bottom wells. Some samples were stimulated with anti-CD3 as described in (A). The number of cells migrated over a 4-h period of exposure were collected and counted. *P* *>* 0.05 when compared *SKAP1*−/− cells to SKAP1+/+ cells in response to CCL21.

Anti-CD3/TCR ligation induces ‘stop-signal’ to arrest T-cell motility [25], while the co-receptor CTLA-4, that increases LFA1 adhesion, can reverse this event [26]. Similarly, increased adhesion via phorbol ester treatment of cells reversed the stop-signal [25]. By contrast, antibodies that increase LFA-1 affinity can arrest cell movement [25]. The strengthening of adhesion can have opposite effects on motility depending on whether it is associated with a concurrent effect on the motility machinery in cells. Our previous studies showed that anti-CD3 can arrest the majority of *SKAP1*−/− T-cells in the absence of chemokine [27]. Some 60% of the *SKAP1*−/− T-cells bound weakly to ICAM1 in the absence of SKAP1, while the remaining population operated in a SKAP1 independent fashion in binding to ICAM1 [27]. We therefore assessed whether anti-CD3 could arrest the motility of *SKAP1*−/− T-cells in presence of SDF-1. SDF-1 can directly activate LFA-1 adhesion but has only a limited ability to reverse the TcR mediated ‘stop-signal’ (i.e. recessive chemokine) [25,28,29]. For this, anti-CD3 was used in plate bound or soluble form in the presence of ICAM-1. Primary resting T-cells were loaded on the upper transwell, while the bottom well was left untreated or contained SDF-1 (Fig. 3). While SDF1 caused a 5–6-fold increase in the migration of SKAP1+/+ T-cells to the lower chamber, this response was markedly inhibited by anti-CD3 treatment in the presence of ICAM-1. Both SKAP1+/+ and *SKAP1*−/− populations were equally affected by anti-CD3, even in the presence of chemokine. Anti-CD3 titration from 0.5 μg/ml to 5.0 μg/ml arrested T-cell migration to SDF-1 in both populations (data not shown). The absence of SKAP1 did therefore not alter the ability of anti-CD3 to induce the ‘stop-signal’ in the presence of SDF1.

As a control, SKAP1+/+ and *SKAP1*−/− T-cells were found to express the same level of CXCR4 on resting cell surface (Fig. 3B, upper FACS profiles). Anti-CD3 treatment reduced the expression levels of CXCR4 to 80% of the untreated control cells in both T-cell populations in terms of the median of fluorescence intensity (MFI) of CXCR4 surface staining (Fig. 3B, right histogram). There was no significant difference in the percentage of CXCR4 positive SKAP1+/+ and *SKAP1*−/− T-cells (Fig. 3B, lower profiles). Overall, these results indicate that anti-CD3 can arrest T-cell motility of both SKAP1+/+ and *SKAP1*−/− T-cells in the presence of SDF-1.

### 3.4 SKAP1 is also not needed for CCL21 induced cell migration of resting T-cells

CCL21 binding to CCR7 receptor also plays an important role in T-cell transmigration through blood vessels, migration in lymph nodes and in T-cell adherence to APCs [1–4]. To assess the role of SKAP1 in response to this chemokine, CCL21 was similarly added to lower wells of a transwell plate and assessed for an ability to attract SKAP1+/+ or *SKAP1*−/− T-cells (Fig. 3C). After a 4-h incubation, a comparable number of T-cells from both populations migrated to the bottom well. The same lack of an effect of SKAP1 deficiency was observed at earlier time points (data not shown). Further, anti-CD3 ligation blocked SKAP1+/+ or *SKAP1*−/− T-cell migration in the presence of a CCL21 gradient (Fig. 3C). These results indicate that SKAP1 is dispensable for CCL21 induced cell migration and cannot reverse anti-CD3 induced ‘stop’ signal in response to CCL21 gradients.

Our findings have previously shown a defect in the ‘inside-out’ pathway induced by the TCR in the activation of LFA-1 adhesion in *SKAP1*−/− T-cells [17,18]. Similar results were obtained with T-cells from ADAP deficient mice that lack both ADAP and SKAP1 [18,22,23]. The SLP-76-ADAP module also plays a central role in LFA-1 induced ‘outside-in’ signalling leading to T-cell polarisation and motility [27]. However, a question remained whether SKAP1 is needed for cell motility induced by chemokines. There are differences (i.e. adenylate cylases) and similarities (i.e. PI 3K) in the nature of the signalling pathways employed by the antigen-receptor and chemokines (SDF-1 and CCL21). Overall, our present findings showed that the motility of primary resting and activated T-cells to SDF1 and CCL21 was unaffected by the absence of SKAP1. This was observed using transwell plates and time-lapse microscopy. Migration in the transwell assay required initial adhesion, as well as the activation of motility to migrate to the lower chamber. The fact there was no difference in the number of cells to migrate to the lower well strongly suggested that sufficient adhesion had been induced in *SKAP1*−/− T-cells to enable subsequent migration in response to the chemokine signal. Similarly, tracings of individual cells confirmed that the velocity and displacement of activated T-cells were the same for SKAP1+/+ and *SKAP1*−/− T-cells. In some experiments, *SKAP1*−/− T-cells showed 5–10% less migration compared to wild-type cells, however, this minor difference was not reproducible over the seven experiments that were conducted. Further, no difference was noted at earlier time points such as 30 min or longer periods of incubation (i.e. up to 6 h). We cannot formally exclude the possibility that there exists a minor difference in the overall affinity or avidly of binding to ICAM1 in *SKAP1*−/− vs. SKAP1+/+ T-cells. However, if a difference does exist; it has no functional consequence as measured by transwell assays and time-lapse microscopy. Similar numbers of T-cells migrated at all times and in response to different concentrations of SDF1. Therefore, within the limits of the conditions used in this study, no apparent difference was noted between the two populations of T-cells. This does not exclude the possible involvement of SKAP1 in different populations of activated T-cells, or under different conditions of adhesion or shear stress. Nevertheless, as an overall effect, it differs markedly from the key role played by SKAP1 in TcR induced LFA-1 adhesion in resting T-cells [27].

No difference was also noted in the ability of SKAP1+/+ and S*KAP1*−/− T-cells to stop in response to TCR ligation in the presence of chemokine. In this assay, a relatively potent signal was delivered to T-cells with soluble anti-CD3 or plate bound anti-CD3 prior to the addition of chemokines. One caveat in this assay is that the subset of primary cells (i.e. >50%) that require SKAP1 expression for TCR induced adherence to ICAM1 may not have been fully measured since they would have bound poorly to plates. Further, T-cells may behave differently in the presence of lower affinity peptide antigen in the presence of a lymph node environment. Nevertheless, using the plate assay, the remaining population of T-cells lacking *SKAP1*−/− failed to show a difference in their response to SDF1 or CCL21. Since chemokines can localise the intermediate affinity of LFA-1 at the leading edge of a migrating cell [13], and high affinity forms of LFA-1 are located at the bottom of cell body [30], one possibility is that while high-avidity clustering of LFA-1 requires SKAP1, the activation of intermediate affinity forms (i.e. induced by chemokines) might suffice to mediate migration to chemokines and to fail to reverse the TCR stop-signal. In the context of motility, another possibility is that chemokines directly activate downstream effectors such as Rap1-RapL without requiring a bridging step provided by SKAP1. Alternatively, it is also possible that the limited expression of related family member SKAP2 (SKAP-Hom or SKAP-related) in T-cells is sufficient to substitute for chemokine, but not TCR induced responses, or that ADAP can compensate for SKAP1 in response to chemokine-induced cell migration. The difference in requirement for the adaptor in responses to TCRs and chemokines could exist to allow for additional junctions of regulation in TCR signalling such as the regulation of the extracellular signal-regulated kinase (ERK) regulation [31,32]. Further studies will be needed to examine whether *SKAP1*−/− T-cells migrate normally to the sites of inflammation/infection.
